# Randomized Clinical Trial on the Use of PHYSTA Freeze-Dried Water Extract of *Eurycoma longifolia* for the Improvement of Quality of Life and Sexual Well-Being in Men

**DOI:** 10.1155/2012/429268

**Published:** 2012-11-01

**Authors:** Shaiful Bahari Ismail, Wan Mohd Zahiruddin Wan Mohammad, Annie George, Nik Hazlina Nik Hussain, Zatul Mufiza Musthapa Kamal, Eckehard Liske

**Affiliations:** ^1^School of Medical Sciences, Universiti Sains Malaysia, Kelantan, 16150 Kubang Kerian, Malaysia; ^2^Department of Science and Clinical Trial, Biotropics Malaysia Berhad, Level 52, Menara TM, Jalan Pantai Baharu, 50672 Kuala Lumpur, Malaysia; ^3^Department of Life Sciences, Technical University of Braunschweig, 38106 Braunschweig, Germany

## Abstract

*Eurycoma longifolia* is reputed as an aphrodisiac and remedy for decreased male libido. A randomized, double-blind, placebo controlled, parallel group study was carried out to investigate the clinical evidence of *E. longifolia* in men. The 12-week study in 109 men between 30 and 55 years of age consisted of either treatment of 300 mg of water extract of *E. longifolia* (Physta) or placebo. Primary endpoints were the Quality of Life investigated by SF-36 questionnaire and Sexual Well-Being investigated by International Index of Erectile Function (IIEF) and Sexual Health Questionnaires (SHQ); Seminal Fluid Analysis (SFA), fat mass and safety profiles. Repeated measures ANOVA analysis was used to compare changes in the endpoints. The *E. longifolia* (EL) group significantly improved in the domain Physical Functioning of SF-36, from baseline to week 12 compared to placebo (*P* = 0.006) and in between group at week 12 (*P* = 0.028). The EL group showed higher scores in the overall Erectile Function domain in IIEF (*P* < 0.001), sexual libido (14% by week 12), SFA- with sperm motility at 44.4%, and semen volume at 18.2% at the end of treatment. Subjects with BMI ≥ 25 kg/m^2^ significantly improved in fat mass lost (*P* = 0.008). All safety parameters were comparable to placebo.

## 1. Introduction

Sexual health is an important issue in quality of life of males and females. In males' sexual well-being, dissatisfaction in sexual life is significantly caused by erectile dysfunction (ED), which is defined as a consistent or recurrent inability of a man to attain and/or maintain penile erection sufficient for sexual activity [1]. Although accurate global values are lacking, almost all reported scientific data on sexual health in men worldwide show that erectile dysfunction is an international problem and prevalence and incidence are influenced by age, with the highest prevalence in men in their 70ties and 80ties of between 50% and 75% [1]. 

Reviews as well as multinational surveys for Asian countries showed the prevalence of self-reported moderate ED in aging males (40–70 years) ranging from 17.7% (Taiwan), 22% (Malaysia), 28.3% (China), 32.2% (Korea), and 34% (Japan) [2–5]. Another survey reported the prevalence of ED in Malay man (mean (SD) age of 58 (7) years) with 32.8% (mild ED), 17.7% (mild to moderate ED), 5.1% (moderate ED), and 14.5% (severe ED) [6]. Due to various reasons, including taboos and cultural restrictions, worldwide most men with ED do not seek for treatment. Nevertheless, ED-sufferers in Asian countries consider traditional herbal medicine as a reliable treatment for improving the overall well-being as was also shown in the Asian MALES study [3, 7, 8].

In Malaysia, one of the most popular herbs, *Eurycoma longifolia* Jack, from the Simaroubaceae family, also known traditionally as Tongkat Ali has been reputed by Malays as a traditional remedy used as an adaptogen for vitality and energy and is well-known for its aphrodisiac activities [9]. Numerous animal studies have scientifically proven an increase in male libido, changes in sexual motivation activities, and results support the aphrodisiac and testosterone-stimulating effects enhancing sexual desire [10–16]. Besides other various biological properties *E. longifolia* also exhibits anxiolytic (antianxiety) effects suggesting medical use for overall physical well-being. 

Traditionally used remedies for enhancing testosterone level in man are (water) extracts of the roots of *E. longifolia*, where a wide range of bioactive compounds have been isolated including phenolic compounds, polypeptides, diterpenoids, alkaloids, quassinoids, and others [9, 17]. In the pursuit of unravelling the underlying mechanisms of actions, it was shown that different fractions of *E. longifolia* root extract on the sexual qualities in male rats, measured using an electrical copulation cage, had in general not much difference in sexual performance, possibly due to the existence of the bioactive compounds in several of the fractions tested [15]. However, the bioactive Eurypeptide with a molecular weight of 4.3 kDa showed reproducible aphrodisiac effects by increasing testosterone levels in Leydig cell cultures, providing the possible explanation to the potency of a so-called phytoandrogen [17].

Clinical trials to support and substantiate the traditional use of *E. longifolia* with its aphrodisiac and ergogenic potency are limited. Previous human investigations have shown that this herb has various beneficial effects on health and can act as an “energy-booster” [18, 19]. Recently, an open label, two-month study in Asian males with hypogonadism and late-onset of hypogonadism (LOH) aged between 28–70 years, showed that a daily intake of 200 mg of *E. longifolia* water extract improved LOH expressed by a significant decrease in Aging Males Symptoms score (AMS) and increased serum testosterone levels [20]. 

Literature-based evidence on the alleged ergogenic potency of (water) extracts of *E. longifolia* in intense strength training programs, general sport activities, for example, endurance running programs and health-related fitness exercises in recreational males and athletes are to some extent inconsistent; however, previous open and placebo-controlled studies with small sample sizes support various positive properties of this herb as an anabolic remedy [18, 21, 22].

The potential of the therapeutic efficacy as well as safety/tolerability of *E. longifolia* can only be demonstrated by robust randomized, controlled trials. Since these data are missing, the present RCT was performed to analyze the improvement of the quality of life, physical performance, and sexual well-being in men during intake of a freeze-dried water extract from the roots of *E. longifolia.*

## 2. Material and Methods

### 2.1. Study Design and Subjects

This 12-week randomized, double-blind, placebo-controlled and parallel group designed trial in Malay males was conducted in accordance with the Guideline for Good Clinical Practice (ICH-6) and Declaration of Helsinki. The study protocol was evaluated and approved by the Human Research and Ethics Committee for Clinical Studies of Universiti Sains Malaysia (USM) (approval date 24 December, 2008). The intervention was carried out from January 2009 (first patient in) to August 2010 (last patient out) at the Clinical Trial Unit (CTU), Hospital Universiti Sains Malaysia (HUSM), Kubang Kerian, Malaysia.

After signed informed consent, healthy married men between 30 years and 55 years of age or those with stable chronic medical illnesses, for example, controlled diabetes mellitus and/or hypertension on monotherapy or low dose combination therapy were included (accepted comedication in obese and overweight subjects, for example, antidiabetic drug metformin and hypertension, for example, antihypertensive drug amlodipine, atenolol, perindopril). Subjects were excluded with major uncontrolled psychiatric disorders, history of alcohol or drug abuse, history of major hematological, renal or hepatic disorder, stroke or myocardial infarction within the last six months, peptic ulcer or bleeding disorder, elevated blood pressure beyond the range of 90/50 to 170/100 mmHg, clinically relevant baseline laboratory abnormality, and/or use of herbal products or drugs that could contain testosterone or any androgenic activity in the last month before start of trial. These products and alcohol were also not permitted during trial. 

Subjects were given the option of withdrawing from the study for any reason at any time or, for example, evidence of intolerance towards *E. longifolia* water extract or unable to comply with the scheduled treatment dose of at least 80% of the dose during the trial period. 

The sample size estimation for this trial was based on data of efficacy studies performed with other traditional herbal medicines since effects of *E. longifolia* extract on the improvement of physical and vitality of adult men were unknown to the date of this study. A study [23] on the effect of *Panax ginseng *in enhancing physical endurance in randomly selected young adults was taken as reference in sample size calculation. The calculation was based on the two-mean formula of between groups (independent *t*) and also prepost trial groups (paired *t*). Level of significance (*α*) of 0.05 and power (1−*β*) of 80% were considered while ratio between trial and control group was taken as 1 (*m* = 1). Considering 10% drop-out rate for the highest calculated sample size of between and within group subject effects, the minimum number of subjects needed in each group arm (verum or placebo) was determined to be 50 with the final sample for this trial was 100 subjects. 

### 2.2. Randomization and Intervention

The subjects adhered to a total of four visit intervals for screening (visit 1, week 1 to week 2), recording of measurements at baseline (visit 2, day 0), at week 6 (visit 3, day 42 ± 3 days), and end of study at week 12 (visit 4, day 84 ± 3 days) followed by a follow-up visit (visit 5 at week 14, via telephone or face to face). At screening (visit 1), clinical examinations and interviews were conducted to select subject who fulfilled the study criteria. Blood samples were collected from the subjects for hematological, renal, and liver parameters such as hemoglobin, white blood count, platelet, creatinine, urea, serum electrolyte, liver enzymes (ALT, ALP, and AST), and Prostatic Specific Antigen (PSA). At baseline (visit 2) all eligible subjects were randomly assigned to the two treatment groups to receive either two capsules of *E. longifolia* water extract or two capsules matching placebo 2 times a day after lunch and dinner for 12 weeks.

During the intervention, each subject consumed four capsules per day containing either verum or placebo. One verum capsule contains as active ingredients 75 mg of the special freeze-dried water extract of *Eurycoma longifolia* root (Physta, Biotropics Malaysia Berhad, Kuala Lumpur, Malaysia) manufactured under Good Manufacturing Process requirements (GMP) with continuous quality control (DER 20 : 1). The total amount of daily intake equals to 300 mg/day herbal extract. Placebo medication corresponded (contained maltodextrin) to the active medication without the herbal extract.

### 2.3. Primary Endpoints

Primary endpoints were taken as the efficacy parameters of Quality of Life (SF-36) questionnaire and physical fitness tests such as flexibility (sit and reach), muscular strength (hand grip; back and leg), muscular endurance (sit-up and push up), and cardiovascular endurance as well as the safety profiles which were measured at each visit after enrollment.

The SF-36 QOL scale, validated for use in Malaysia [24], includes questions classified in 8 domains/dimensions: physical functioning, role physical, bodily-pain, social functioning, mental health, role emotional, vitality, and general health perception. 

Each session of the physical fitness tests contained of a battery of different types of tests so that various physiological and muscular parameters could be activated/stimulated, finally leading to an improvement of these fitness activities: flexibility—(type of test: sit and reach), muscular strength (hand grip; back and leg), muscular endurance (sit-up and push up), body composition (Bioelectrical Impedance Analysis), and cardiovascular endurance/cardiorespiratory fitness. All these tests were carried out on the same day for each participant and the duration took approximately two hours for each session of five to eight subjects. 

The safety assessments reflected the documented Adverse Events (AE, description, severity, causality to treatment as assessed by the investigators), physical examination, clinical and laboratory measures including blood urea serum electrolytes (BUSE), creatinine, Serum Glutamate-Oxalacetate Transaminase (AST), Serum Glutamate-Pyruvate Transaminase (ALT), alkaline phosphatase (ALP), total protein, albumin, globulin and bilirubin, Prostatic Specific Antigen, glucose, uric acid, lipid profile, full blood count, and the ratio testosterone : epitestosterone. 

### 2.4. Secondary Endpoints

The secondary endpoints were assessed at baseline and after 6 weeks and 12 weeks of treatment: Sexual Health Questionnaires (SHQ) and International Index of Erectile Function (IIEF-15), hormonal profiles, that is, free testosterone, serum total testosterone, Insulin-like Growth Factor 1 (IGF-1), dehydroepiandrosterone sulfate (DHEA-SO_4_), sex hormone binding globulin (SHBG), Seminal Fluid Analysis (SFA, using standardized methods for collection [25]), and fat loss by Dual Energy X-ray Absorptiometry scan (DEXA) for body fat composition. 

The SHQ used in this study contains questions divided into two domains “Sexual Libido” and “Sexual Satisfaction”. The IIEF-scale which is useful to evaluate erectile dysfunction of a population consists of 15 questions divided into five domains: “Erectile Function”, “Orgasmic Function”, “Sexual desire”, “Intercourse Satisfaction”, and “Overall Satisfaction”. The scores correspond to clinical interpretations ranging from “severe” to “no dysfunction”. The IIEF-scale is culturally adapted and validated for use in Malaysia [26].

Blood tests were analyzed at the Gribbles's clinical laboratories (Petaling Jaya, Selangor, Malaysia). DEXA scan and SFA (single lab technician) were conducted at Hospital Universiti Sains Malaysia (HUSM). Physical fitness tests were conducted by Sport Science Lab personnel at HUSM.

### 2.5. Statistical Analysis

The main aim of the statistical analysis was to explore the safety profiles and to show the effectiveness of the trial drug measured by changes of the parameters in the *E. longifolia* group (primary and secondary outcomes) versus placebo at week 6 and/or end of treatment after 12 weeks in comparison to baseline values by using the Intention-to-Treat (ITT) analysis approach. Only data sets from subjects who took at least one dose of study drug and had at least one postbaseline efficacy evaluation were included. Missing values were handled by last observation carried forward (LOCF). As a secondary approach, data were analyzed from the Per Protocol (PP) population following subjects who completed all of the three visits (V2, V3, V4) without violation or major deviations and who were at least 80% compliant in taking medication.

Data was explored and descriptive statistical analysis was conducted by variables especially on the safety profiles. Mean (SD, standard deviation) was described for continuous variables of normality distributed data and median (IQR, inter quartile range) for nonnormal distribution, while frequency and percent (%) were described for categorical data. Independent *t*-test, chi-square (*χ*2) analyses, and nonparametric Mann-Whitney test (as appropriate) were used for baseline comparison of the two randomized groups. Repeated measure ANOVA (mixed design) was conducted for changes over time for the continuous outcomes (within groups difference), between-groups differences and also across the two treatment groups (i.e, a group by time interaction), from baseline to week 6 and week 12. Further procedure was followed when repeated measures ANOVA (within subject effects) were found to be statistically significant on primary continuous outcomes using paired *t*-tests with Bonferroni pair wise comparisons between time periods. Adjustment of the outcome parameters for certain confounders or covariates (including the baseline differences) was carried out using analysis of covariance (ANCOVA). 

Further efficacy analysis on selected outcome parameters at significance level *α* = 0.10 (two-tailed) (10% level of significance) was also conducted if no significant result of initial analysis seen at *α* = 0.05 Additionally, an age-dependent subgroup analysis was done for age groups of 30 years to 44 years and 45 years to 55 years. 

All data were documented in the case report forms (CRF). Data entry was done at Clinical Trial Unit (CTU), School of Medical Sciences, Universiti Sains Malaysia USM. Results from the trial were analysed and stored at School of Medical Sciences, Universiti Sains Malaysia. The statistical analysis was performed using Statistical Package Social Sciences, SPSS (PASW 18.0) under license of USM, Malaysia.

## 3. Results

**Figure 1:**
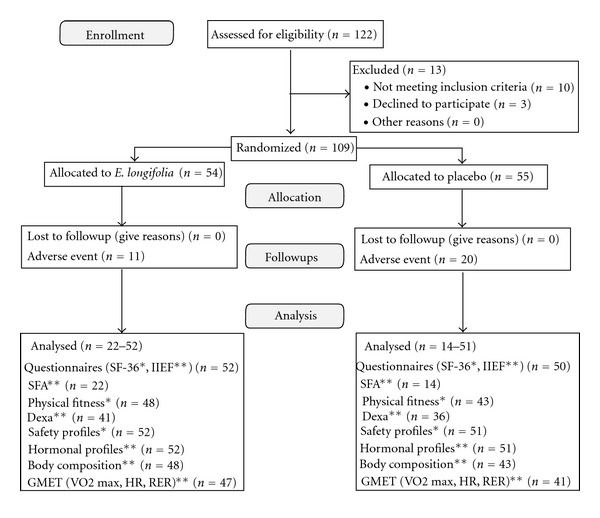
Number of subjects as in the Intention-to-Treat analysis datasets by primary* and secondary** outcomes. Subjects were omitted for analysis if they have no baseline data and/or without at least one postbaseline evaluation at week-6 or week-12. For SFA, only 36 subjects who turned up at both visit 2 and visit 4 were included for efficacy analysis. For safety profiles, the full dataset included all subjects who took at least one dose of study medication and had at least one postbaseline efficacy evaluation.

A total of 122 male subjects were screened for eligibility and 109 subjects were randomized to* E. longifolia* and placebo medication. Subjects were omitted for analysis if they had no baseline data and/or without at least one postbaseline evaluation at week 6 or week 12. The Intention-to-Treat (ITT) population is shown in Figure 1. This study focused on the ITT-approach for primary efficacy analysis. Per Protocol (PP) analysis was added as secondary approach derived from the ITT-population, complying with the protocol sufficiently and also excluding any major protocol violations. The number of PP subjects in both groups is similar to the dataset in the ITT analysis and due to this, the results are similar. For safety profiles, the full analysis set included all subjects who took at least one dose of study drug and had at least one postbaseline efficacy evaluation.

### 3.1. Subject Characteristics

Baseline comparisons on demographic and physical examination data on study participants between randomized groups of *E. longifolia* and placebo show no statistical difference (Table 1). The Body Mass Index BMI values for all subjects range from 19.0 kg/m^2^ to 40.1 kg/m^2^ with mean (SD) of 26.14 (3.71) kg/m^2^. The BMI proportions between normal (<25), overweight (≥25 to <30), and obese (≥30) were 36.7% (40/109 subjects), 53.21% (58/109), and 10.09% (11/109), respectively. Regarding the Fasting Blood Sugar the values range from 4.3 mmol/L to 12.1 mmol/L, with 83.33% (90/108) below the value of 5.5 mmol/L. The remaining proportion had elevated glucose levels. Blood pressure ranged between SBP 90 mmHg to 160 mmHg and DBP from 59 mmHg to 94 mmHg; approximately 45% (49/109) of the subjects showed values above SBP 120 mmHg and 5.5% (5/109) had values above SBP 140 mmHg. 

There is no significant difference between groups with respect to hematological or clinical blood chemistry parameters including Fasting Blood Sugar, liver function, full blood counts, Prostate Specific Antigen (PSA), lipid profile, and hormone profile except for total testosterone, whereby subjects in the placebo group had significantly higher total testosterone values (mean 18.8 (4.6) nmol/L) compared to *E. longifolia* group (16.5 (5.8) nmol/L) (*P* = 0.026). SHBG levels are slightly elevated in the placebo (30.6 (10.3) nmmol/L) compared to the herbal group (26.8 (11.5) nmmol/L) (*P* = 0.073). Seminal Fluid Analysis (SFA) and DEXA (trunk, legs and arm & legs) show no difference between groups at baseline.

### 3.2. Primary Efficacy Endpoints

#### 3.2.1. Quality of Life (SF-36)

For the ITT-analysis, data from 102 subjects (52 *E. longifolia* and 50 placebo) were used including the three cases (all placebo) with missing data on week 12, treated with LOCF principle. The general analysis shows no overall significant mean differences over time between *E. longifolia* and placebo. However, significant improvements and group differences as well as trends are observed in various items in several domains of the Quality of Life SF-36 scale.

From the eight domains in the SF-36 forms, the domain “physical functioning” (9 items on moderate and vigorous activities, climbing, bending and kneeling, walking, and bathing/dressing), “role physical”, and “vitality” showed significant improvements. 

**Figure 2:**
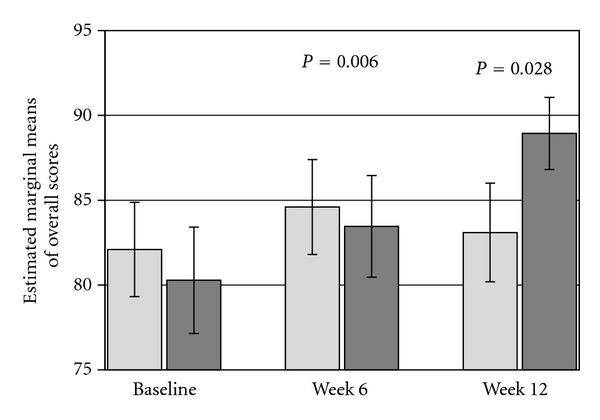
Mean values of overall scores (±SEM) of Physical Functioning domain from baseline to week 12 for *E. longifolia* (The dark grey column, *n* = 52) and placebo (the grey column, *n* = 50).

In the *E. longifolia* group, there was a significant improvement of overall scores of Physical Functioning domain from baseline to week 12 (*P* = 0.006) as compared to placebo (Figure 2). In addition, the difference between the two groups changes over time and the scores among subjects who took the herbal product are significantly higher than placebo at week 12 (*P* = 0.028). No age-related effect is observed. The score values of the single items from the Physical Functioning domain for herbal and placebo groups correspond to the clinical interpretation that the activities during a typical day “yes, limited a little” to “no, not limited at all”. 

On Reported Health Transition *(“Compared to a year ago, how would you rate your health in general now?”), *an overall significant change from baseline to end of study at week 12 was observed in the herbal group as compared to placebo (*P* = 0.009). In contrast, no significant changes were seen in placebo group. Analysis by age stratification (30 yrs to 44 yrs and 45 yrs to 55 yrs) showed no significant changes or differences between groups over time, however, an increasing trend was seen in younger males (30 yrs to 44 yrs). The mean score values for subjects in both groups corresponded closely to the answer “about the same as one year ago”. 

In other SF-36 domains, increasing trends are seen in role physical, vitality, and general health in *E. longifolia* subjects but the changes were not statistically significant.

#### 3.2.2. Physical Fitness

Eighteen subjects (12 placebo and 6 *EL*) were not included in the analysis due to no (at least one) postbaseline evaluation. Thus, 91 subjects (43 placebo and 48 EL) were analyzed including three cases (all placebo) with missing data treated as LOCF principle.

Comparison on each physical test outcomes between *E. longifolia *and placebo groups at baseline and each follow-up visits shows no significant difference. Regarding the “Back & Legs test”, subjects in the herbal group show an overall significant improvement during treatment from baseline to week 12 (*P* = 0.001). However, a similar trend was also shown in the placebo group. Changes in other physical tests including cardiovascular endurance are not significant.

### 3.3. Secondary Efficacy Endpoints 

#### 3.3.1. Erectile Function (IIEF)

For the analysis, 102 subjects (52 EL and 50 placebo) were included where three cases (all on placebo) with missing data on week 12 were treated with LOCF principle.

For all analyzed subjects the scores in the five domains at baseline correspond to clinical interpretation as having “no” or “mild” “erectile dysfunction”.

In the herbal group the overall Erectile Function score increases significantly from baseline to week 12 as compared to placebo (*P* < 0.001), indicating a meaningful improvement on erectile functioning in subjects on *E. longifolia, *however still within the score range of “no dysfunction” at score values of 25 to 30 (mean ± SE; Day 0: 25.365 ± 0.479; V4: 26.788 ± 0.443). Age-related effects are not seen in both herbal and placebo groups. 

Analysis of individual questionnaire items under the Erectile Function domain reveals for item Q1 “*Over the past 4 weeks, how often were you able to get an erection during sexual activity?*” a significant and age-independent increase by 8.7% from baseline to end of the study after 12 weeks within the range of “most times (more than half the time)” to “almost always or always” (*P* = 0.001) were observed in subjects on *E. longifolia*, whereas there was no significant difference in placebo group. Similar results can be seen for item Q3 “*Over the past 4 weeks, when you attempted sexual intercourse, how often were you able to penetrate (enter) your partner?*”, with a significant difference (increase by 7.2%) in *E. longifolia* subjects from baseline to week 12 (*P* = 0.004). The placebo group showed no significant differences and the effect under *E. longifolia* is independent of age.

In the other IIEF domains no significant changes were observed between both groups.

#### 3.3.2. Sexual Health Questionnaire (SHQ)

For the analysis, 102 subjects (52 EL and 50 placebo) were included where three cases (all placebo) with missing data on week 12 were treated with LOCF principle. 

At baseline, the analyzed subjects had in both groups score values of approximately 67% to 73% of the maximum score points (total score range min–max: 3 to 15 for sexual libido; total score range min–max: 5 to 22 for sexual satisfaction). No significant difference on Sexual Libido and Sexual Satisfaction mean scores is detected between *E. longifolia* and placebo at baseline, week 6 and week 12. 

However, the overall Sexual Libido score for subjects in the herbal group significantly increased between week 6 and week 12 as compared to placebo (*P* < 0.001) after first declining from baseline to week 6, with similar results for placebo. Looking at selected items, the score difference of item IA of the Sexual Libido domain *“Over the last 4 weeks, how is your interest towards sexual relationship?”,* increased significantly by 8.4% within the range of “moderate” (score value 3) to “high” (score 4) (*P* = 0.009) between baseline (mean ± SE; 3.211 ± 0.084) and week 12 (3.481 ± 0.093) for subjects in the herbal group. Values of item IB “*Over the last 4 weeks, as compared to the previous 4 weeks, how is your interest towards sexual relationship*?”, significantly improved by an 10.8% increase from baseline (score 3.058 ± 0.075) and week 6 (3.385 ± 0.092; *P* = 0.003) and between baseline and week 12 (3.500 ± 0.097) by 14.4%, ranging between the answers “no change” (score 3) to “slightly increasing” (score 4) (*P* < 0.001). Similarly, the score of item IC “*Over the last 4 weeks, as compared to the previous 4 weeks, have you had sexual dreams?*”, significance is reached between baseline and week 12 by an elevated score of +24.4% (*P* = 0.005) corresponding to improvement within the range from “no” dreams to “1-2 times” dreams. 

Regarding the overall Sexual Satisfaction scores, the *E. longifolia* group shows a significant change between baseline and week 12 due to an increase score by 7.2% (*P* = 0.001). For the selected item IIB of the Sexual Satisfaction domain “*Over the last 4 weeks, as compared to the previous 4 weeks, the frequency of your sexual relationship is increased?*”, there is a significant increase score by 14.4% (baseline—week 6; *P* < 0.001) and 17.1% from baseline (2.923 ± 0.066) to week 12 (3.423 ± 0.093; *P* < 0.001). The frequency of sexual relationship improved from “decreased moderately” (score 2) at baseline to “neither increased nor decreased” (score 3) and “increased moderately” (score 4) at the end of the study.

### 3.4. Other Primary and Secondary Endpoints

#### 3.4.1. Fat Mass Loss (by DEXA Scan)

DEXA scan was performed in 97 subjects (49 placebo and 48 EL) for ITT analysis (8 LOCF from baseline and 12 LOCF from week 6) and considered as a secondary endpoint.

No significant difference between *E. longifolia* and placebo groups in fat mass ratio at baseline, week 6 and week 12 could be evaluated after start of the trial. However, there is a reducing trend in overweight subjects with BMI > 25 kg/m^2^ of fat mass loss (trunk area) during herbal intake as compared to placebo (*P* = 0.008).

#### 3.4.2. Hormonal Profiles

Throughout the study no significant changes among the *E. longifolia* subjects or differences to placebo are seen in hormonal profiles (testosterones, IGF-1, SHBG, DHEASO4). Analysis of covariance (ANCOVA) shows that there are no differences between groups at week 6 and week 12 in total testosterone and free testosterone after adjusting to their baseline values. During the trial the mean values of total testosterone level are in the range of 15.079 nmol/L to 16.604 nmol/L in subjects on the herbal product and in the placebo group these values range from 18.120 nmol/ to 19.051 nmol/L.

#### 3.4.3. Seminal Fluid Analysis (SFA)

Analysis was performed in 36 subjects (22 EL and 14 placebo) who have participated in both SFA examinations (at baseline V2 and week 12 V4).

Between group comparisons on all SFA parameters (volume, count, % motility, and normal oval) at baseline and after week 12 showed no significant difference at 5% and 10% level of significance. The changes and differences between groups with regard to time were not significant even after controlling the baseline values, age, smoking status, number of children, and marriage duration as shown by ANCOVA analysis.

**Figure 3:**
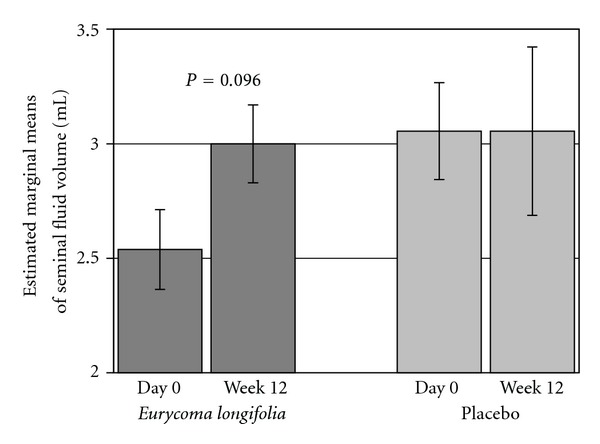
Mean seminal fluid volumes (mL; ±SEM) for subjects with low baseline (Day 0) values with cutoff 3.5 mL after 12 weeks intake of *E. longifolia* (The dark grey column, *n* = 13) and placebo (the grey column, *n* = 9).

Total Seminal Fluid Analysis volume shows a slight increasing trend during intake of the herbal product (baseline to V2: 3.273 mL and V4: 3.454 mL) in contrast to stable values or a declining trend for placebo subjects (V2: 3.714 mL, V4: 3.607 mL). However, subgroup analysis with different baseline values reveals relevant and significant improvement of SFA volume in subjects who took* E. longifolia *(Figure 3). The results for subjects in the herbal group with lower starting values at baseline (taken the median value 3.5 mL as the cutoff point) show statistical difference by a mean increase of 18.2% from a volume of 2.54 mL at baseline to a volume of 3.00 mL after 12 weeks (*P* = 0.096, *n* = 13). In contrast, there was no change in mean values of SFA volume over time, for subjects in the placebo group (baseline: 3.056 mL, V4: 3.056 mL) with lower starting values. 

**Figure 4:**
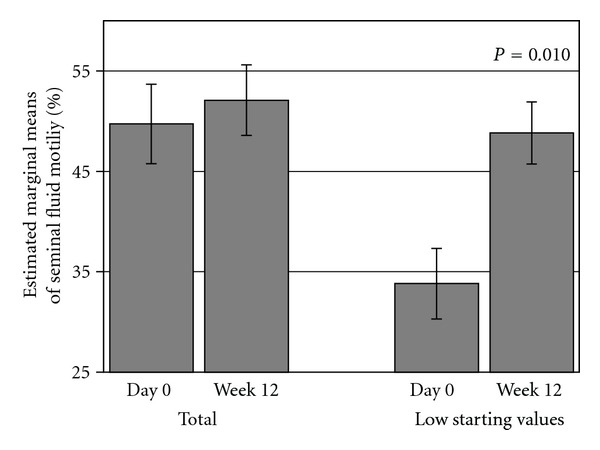
Mean motility (%) (±SEM) for all subjects (total, *n* = 22) and subgroup of subjects (*n* = 11) with low starting values at baseline (Day 0) and after 12-week treatment with *E. longifolia. *

Similar results were obtained from a subgroup analysis of SFA motility parameters, when their baseline values were divided between low and high starting points (taking the median value as the cutoff point) (Figure 4). The results demonstrate statistical improvement in *E. longifolia *subjects (*n* = 11) with lower baseline values by an average increase of 44.4% (*P* = 0.010) from baseline value of 33.8% to 48.8% sperm motility after 12 weeks treatment with no significance in interaction in placebo group (baseline: 36.250%, V4: 44.625%; *P* = 0.252).

There is also an increasing trend in the SFA sperm Ova-shaped parameters in the herbal group as compared to placebo for (+4.3%, *P* = 0.009). 

#### 3.4.4. Safety Evaluation

**Clinical, Laboratory, and Physical Examinations**

All safety parameters obtained from clinical, laboratory, and physical examinations reflect no significant difference between *E. longifolia* and placebo groups at baseline, week 6 and end of the study at week 12 (*P* > 0.05). However, there are significant changes in some renal functions parameters (uric acid, serum creatinine, and potassium) in both herbal and placebo groups but without any clinical relevance.

**Adverse Events**

Two Serious Adverse Events (SAEs) occurred in 1 subject at the end of trial (V4) in the herbal group and were not related to treatment (hospitalization: low back pain; liposome). A total of 31 Adverse Events (AEs) were recorded in 26 subjects: 20 AEs in 15 subjects from the placebo group and 11 AEs in 11 subjects during intake of *E. longifolia *with mild to moderate severity (Table 2). All AEs were assessed as “unlikely” when relating to the treatment except for 1 AE in the placebo group (“headache”) which was assessed as “probable”.

## 4. Discussion 

*Eurycoma longifolia* is a well-known traditional herb in South-East Asian countries and used as a popular medicinal product in daily life in Malaysia. Extracts of this herb are considered in the management of male's sexual dysfunction and health-related quality of life. Besides the traditional evidence, robust data from randomized controlled clinical trials on a larger study population on the efficacy and safety of water extracts of *E. longifolia* are lacking. The present randomized controlled clinical trial in 109 male subjects aged 30 years to 55 years for 12 weeks on a special aqueous *Eurycoma longifolia *root extract 300 mg/daily or placebo was conducted as a result. 

During daily intake of the herbal extract, the quality of life, physical fitness, erectile functioning and sexual health, sexual libido, and sexual satisfaction improved in various domains over time and several items of these questionnaires showed meaningful changes and statistically significant trends compared to placebo. In spite of score values at approximately 70% of the maximum attainable scores at baseline, scattered but remarkable efficacy results were seen in quality of life, physical fitness, and sexual well-being in men treated with *E. longifolia*, although assessment over time was limited due to the so-called ceiling effect already established at the beginning (baseline) of the trial. 

This trial provided important results on males' erectile functioning and sexual health, substantiating the traditional use of *E. longifolia* for the treatment of erectile dysfunction (ED) and overall sexual well-being. The International Index of Erectile Function (IIEF-15) scale was used to evaluate ED within the study population. The total score showed at baseline for subjects in both groups values within the range of either “mild dysfunction” or “no dysfunction”. However, comparison of the two treatment groups resulted in a significant increase of total IIEF score in subjects who took the herbal product from baseline to end of trial after 12 weeks, compared to placebo. Selected IIEF items on erection during sexual activity or frequency of penetration during intercourse, increased significantly during a 12-week intake of *E. longifolia* when compared to placebo. When using the Sexual Health Questionnaire (SHQ) the preceding observations are consistent with a significant increase of interest towards sexual relationship, more frequent sexual dreams and sexual relationship as well as satisfaction improvement, all in favor of the herbal treatment. 

These data support the attitudes of Malaysian men of preventing or treating erectile dysfunction with traditional herbal medicine including Tongkat Ali (*E. longifolia*), which is also known to improve overall well-being [3] and is also demonstrated by results from a recent open clinical study [20]. In that study, in patients (*n* = 64/76 Malaysian) with low-onset of hypogonadism with mean age 51 years (range 28 yrs–70 yrs), 200 mg/daily of the same aqueous *E. longifolia* root extract as used in the present study, significantly improved Aging Male Symptoms (AMS-score) from score values corresponding to “mild complaints” at baseline to “no complaints” after one month of intake (*P* < 0.001). This was also significantly correlated with an increase in serum testosterone levels. 

In the present study, Seminal Fluid Analysis (SFA) showed remarkable but unexpected results in primarily healthy subjects who took* E. longifolia *extract for three months since the testosterone (total and free) levels at baseline were considered to be in the normal range with a marginal increase at end of the study. The volume of seminal fluid and the sperm motility for a subgroup with lower starting values at baseline revealed significant increases at end of trial when treated with* E. longifolia *(volume + 18.2%; motility + 44.4%), whereas no changes were observed in the placebo group. Increasing trends were observed also in the sperm-shape parameters where more normal-shaped compared to abnormal-shaped sperms were observed. 

*E. longifolia* is known to improve males' fertility. In a recent open clinical study performed by Bin and Imran, 2010 [27], in young males (mean age 32.7 yrs) with a history of several years of idiopathic infertility, 200 mg/day of the same *E. longifolia* extract used in the present trial improved significantly the semen parameters over a 3-month (*n* = 75) to 9-month (*n* = 17) treatment period. Compared to baseline, sperm volume increased significantly up to 9 months (+19%), whereas sperm concentration (+54%) and motility (+12%) significantly peaked after 3-month treatment with *E. longifolia*. The corresponding values of the sperm parameters are almost similar to the ones observed in the present randomized trial when analysed with lower starting point: sperm volume at baseline and after 3-month treatment: Bin and Imran [27]: mean ± SE  2.95 mL ± 0.14 mL and 2.96 mL ± 0.13 mL, respectively; this trial: mean 2.54 ml and 3.00 ml, respectively. In the Bin and Imran 2010 study [27], this value was first reached after 9-month treatment. Sperm motility at baseline and after three months: Bin and Imran [27]: mean ± SE  44.68% ± 2.44% and 49.99% ± 2.81%, respectively; this trial: mean 33.8% and 48.8%, respectively. Comparing these data it seems that in the present trial the effect was more prominent, leading to the assumption that the higher dose of 300 mg per day of this *E. longifolia *root extract is more effectively in semen parameters.

It is so far known that the traditionally used water extracts of the root of *E. longifolia* are described as being safe. This is supported by the present trial showing no differences in all analyzed safety parameters and Adverse Events (AEs) monitoring as compared to placebo. 

## 5. Conclusion

For the first time a placebo-controlled clinical trial with 109 randomized male subjects demonstrated for a freeze-dried water extract of the roots of *Eurycoma longifolia *(Physta), significant improvements in libido, sexual performance, satisfaction, and physicial functioning. *E. longifolia* has a strong impact on seminal fluid parameters, for example, semen volume and sperm motility. The daily dose of 300 mg of *E. longifolia* extract for three months is well-tolerated and safe compared to placebo. 

## Figures and Tables

**Table 1:** Baseline comparisons on demographic and physical examination data on study participants between randomized groups of *E.* *longifolia* and placebo. Results are expressed as mean ± SD (standard deviation) unless mentioned.

Variables	*E. * *longifolia* (*n* = 54)	Placebo (*n* = 55)	*P* value*
Demographic characteristics			

Age (years)	43.6 (6.52)	42.8 (6.73)	0.553
Educational level (*n*, %)			
Primary	4 (7.5)	0 (0.0)	
Secondary	43 (81.1)	49 (89.1)	0.113^‡^
Tertiary	6 (11.3)	6 (10.9)	
Ethnicity (*n*, %)			
Malay	53 (98.1)	53 (96.4)	0.609^‡‡^
Non-Malay	1 (1.9)	2 (3.6)	
Duration of married (mean years, SD)	16.9 (8.02)	17.2 (7.27)	0.842
No. of children (median, range)	4 (0–13)	4 (0–10)	0.718**
Smoking	18 (33.3)	24 (43.6)	0.609^‡^

Physical examinations			

Systolic blood pressure (mmHg)	118.1 (12.9)	118.9 (13.1)	0.750
Diastolic blood pressure (mmHg)	76.1 (8.9)	74.2 (8.6)	0.259
Height (meter)	1.65 (0.05)	1.67 (0.06)	0.171
Body weight (kg)	71.9 (10.5)	71.9 (11.1)	0.975
Waist circumference (cm)	86.0 (11.5)	85.3 (13.8)	0.757

*Independent *t*-test; ^‡^chi-square test, ^‡‡^Fisher exact test; **Mann-Whitney *U* test.

**Table 2:** Incidence of adverse events (AE) observed during the study. The number of AEs recorded for subjects on *E.* *longifolia* and placebo with assessed causality to treatment.

Adverse event	*Eurycoma * *longifolia *	Placebo	Causality
Headache	0	1	Probable
URTI with viral fever	1	0	Unlikely
URTIS	3	5	Unlikely
Generalised body ache	1	0	Unlikely
Conjunctivitis	1	2	Unlikely
Infected chalazion	1	0	Unlikely
Ankle pain	1	1	Unlikely
Epigastric pain	0	1	Unlikely
Lumbar pain	0	1	Unlikely
Archilles tendinitis	1	0	Unlikely
MVA with abrasion	0	1	Unlikely
Pneumonia	0	1	Unlikely
Medial meniscus injury	0	1	Unlikely
Foot pain	0	3	Unlikely
Herpes zoster	1	0	Unlikely
R index finger pain	1	0	Unlikely
Muscle sprain	0	1	Unlikely
Eye discomfort	0	1	Unlikely
Age	0	1	Unlikely

**Total** **AEs**	**11**	**20**	

**Total subjects**	**11**	**15**	
